# A high-throughput amplicon-based method for estimating outcrossing rates

**DOI:** 10.1186/s13007-019-0433-9

**Published:** 2019-05-18

**Authors:** Friederike Jantzen, Natalia Wozniak, Christian Kappel, Adrien Sicard, Michael Lenhard

**Affiliations:** 1 Institute for Biochemistry and Biology, University of Potsdam, Karl-Liebknecht-Str. 24-25, House 26, 14476 Potsdam-Golm, Germany; 2 Present Address: Molecular Genetics and Physiology of Plants, Faculty of Biology and Biotechnology, Ruhr University Bochum, Universitätsstraße 150, Building ND North, 44801 Bochum, Germany; 3 Present Address: Department of Plant Biology, Swedish University of Agricultural, Sciences and Linnean Center for Plant Biology, Uppsala, Sweden

**Keywords:** Outcrossing, Mixed mating, Outcrossing rate, *Capsella*, Amplicon sequencing

## Abstract

**Background:**

The outcrossing rate is a key determinant of the population-genetic structure of species and their long-term evolutionary trajectories. However, determining the outcrossing rate using current methods based on PCR-genotyping individual offspring of focal plants for multiple polymorphic markers is laborious and time-consuming.

**Results:**

We have developed an amplicon-based, high-throughput enabled method for estimating the outcrossing rate and have applied this to an example of scented versus non-scented *Capsella* (Shepherd’s Purse) genotypes. Our results show that the method is able to robustly capture differences in outcrossing rates. They also highlight potential biases in the estimates resulting from differential haplotype sharing of the focal plants with the pollen-donor population at individual amplicons.

**Conclusions:**

This novel method for estimating outcrossing rates will allow determining this key population-genetic parameter with high-throughput across many genotypes in a population, enabling studies into the genetic determinants of successful pollinator attraction and outcrossing.

**Electronic supplementary material:**

The online version of this article (10.1186/s13007-019-0433-9) contains supplementary material, which is available to authorized users.

## Background

The rate at which individuals in a population outcross has a major impact on the genetic structure of the population and its responses to natural selection [1, 2]. While outcrossing maximizes the heterozygosity in a population, selfing or inbreeding between relatives increases homozygosity. This, in turn, has a number of consequences, such as the phenotypic expression of recessive deleterious mutations, also known as inbreeding depression [3], and a reduced rate of effective recombination, as crossing over between homozygous chromosomes does not lead to the formation of genetically recombinant gametes [4]. Over time, such a reduced effective rate of recombination leads to an increased length of haplotype blocks in linkage disequilibrium and of linked selection [4]. In addition, inbreeding reduces the effective population size, and as a result, the relative importance of genetic drift increases compared to that of selection [5]. The reduced efficacy of purifying selection also increases the risk of fixation of deleterious mutations and influences species extinction rates [6]. Thus, the outcrossing rate is a key determinant of several population-genetic parameters with a major influence on long-term evolutionary trajectories of populations [2, 7].

In contrast to animals, where outbreeding enforced by dioecy is seen in the majority of species [8], most flowering plants are hermaphrodites [9]. While many plant lineages have evolved genetic self-incompatibility and other mechanisms to enforce or promote outbreeding, mixed mating is very common in plants [10]. In mixed-mating species, a fraction of the progeny of a plant is derived from selfing, while the rest is the result of outbreeding. Therefore, estimating the outcrossing rate of plants in a population is an important aspect of studies in plant reproductive systems.

Classically, the outcrossing rate is estimated by genotyping a large number of progeny individuals from a focal individual for several microsatellite or SNP markers and determining the fraction of genotypes that cannot have been produced by selfing at each marker [11, 12]. From these data, rates of outcrossing and other parameters of the breeding system can then be estimated [13, 14]. While this approach can provide a rich and nuanced picture of the breeding system in a population, it is laborious and thus not readily amenable to be used in a high-throughput manner. Examples for questions that require such a high-throughput approach would be the following. How does the breeding system of a species depend on different environmental conditions? Are rates of outcrossing stable within a population over different years? And how does variation in floral characteristics influence outbreeding rates? Answering this kind of question requires estimating outcrossing rates for a large number of focal individuals, which would be prohibitive when done by genotyping many progeny individuals per focal plant.

Concrete examples for the last of the three mentioned question are studies to determine the relevance of different traits presumed to help in pollinator attraction, such as large and showy petals, emission of floral scent, and nectar amount and composition [15]. These traits often undergo large changes, when the breeding system changes from predominant outbreeding to selfing [16]. This transition is generally accompanied by the evolution of the so-called selfing syndrome, comprising a reduction in flower size, especially that of petals, in scent and nectar production and in the ratio of pollen to ovules per flower. One example where the genetic basis of the evolution of the selfing syndrome is being studied is the genus *Capsella* [17, 18]. This genus contains three diploid species, two of which (*C. rubella* and *C. orientalis*) represent independently derived selfers that have diverged from an outbreeding ancestor represented by present-day *C. grandiflora* between 100,000 and 200,000 years ago and between one and two million years ago, respectively [19–21]. Several loci have by now been identified that have contributed to the reduction in petal size and in floral scent in *C. rubella* compared to *C. grandiflora* [22–24]. While this is starting to shed light on the molecular basis and evolutionary history of selfing-syndrome traits, understanding the ecological consequences of changes in presumed pollinator-attraction traits remains a major challenge. That said, several key biological materials have become available as part of the process of gene identification - such as quasi-isogenic lines (qILs) that only segregate for a very small chromosomal segment containing a causal gene, but are essentially isogenic otherwise—that will enable rigorously testing the effect of a given trait change on the interaction with pollinators and herbivores, including on the outcrossing rate. However, as outlined above, such studies would greatly benefit from a high-throughput method for estimating outcrossing rates from many individual plants differing in a gene and thus a trait of interest.

Against this background of work on *Capsella*, we set out to establish and evaluate a high-throughput compatible method for estimating and comparing outcrossing rates. This method is based on Illumina sequencing of PCR fragments amplified from pooled progeny individuals of a plant and estimating the outcrossing rate from the frequency of non-maternal haplotypes.

## Methods

### Reagents

- Gibberellic acid 4 + 7 (Duchefa Biochemie)
- Ethanol (Carl Roth)
- Paper bags for bagging plants (HERA)
- Bird protection mesh (mesh size 25 mm) (Zill GmbH Co. KG)
- Insect protection mesh (mesh size 0.6 mm) (Grow it)
- 2 ml and 1.5 ml tubes
- 96 well PCR plates (Sarstedt)
- Foil/lids to seal plates
- 384 Well Lightcycler plates (Sarstedt)
- Adhesive Optical film (Biozym Scientific)
- Nuclease-free water
- Liquid nitrogen
- Quiagen DNeasy Plant Mini Kit (Quiagen)
- AMPure XP beads (Beckmann Coulter)
- Magnetic stand (Applied Biosystems)
- KAPA HiFi Hotstart PCR Kit with dNTPs (Roche)
- DMSO (Carl Roth)
- ROX solution (ThermoFisher Scientific)
- SYBR Green I Nucleic acid stain (Sigma-Aldrich)
- TE buffer (10 mM Tris–HCl, pH 7.5)
- QuantiFluor dsDNA System (Promega)
- Tapestation reagents (Agilent Technologies)
- Qubit reagents (ThermoFisher Scientific)
- NextSeq Reagent Kit (Illumina)

### Equipment

- Mortar and Pistil
- Multichannel and single-channel pipettes
- LightCycler 480 II (or similar) (Roche)
- Mastercycler nexus (or similar) (Eppendorf)
- Speedvac RVC 2-18 (Martin Christ Gefriertrocknungsanlagen)
- NextSeq (Illumina)
- Centrifuges (for plates and tubes)
- 2200 TapeStation (Agilent Technologies)
- Qubit 2.0 (ThermoFisher Scientific)

### Plant material and growth conditions

**Fig. 1:**
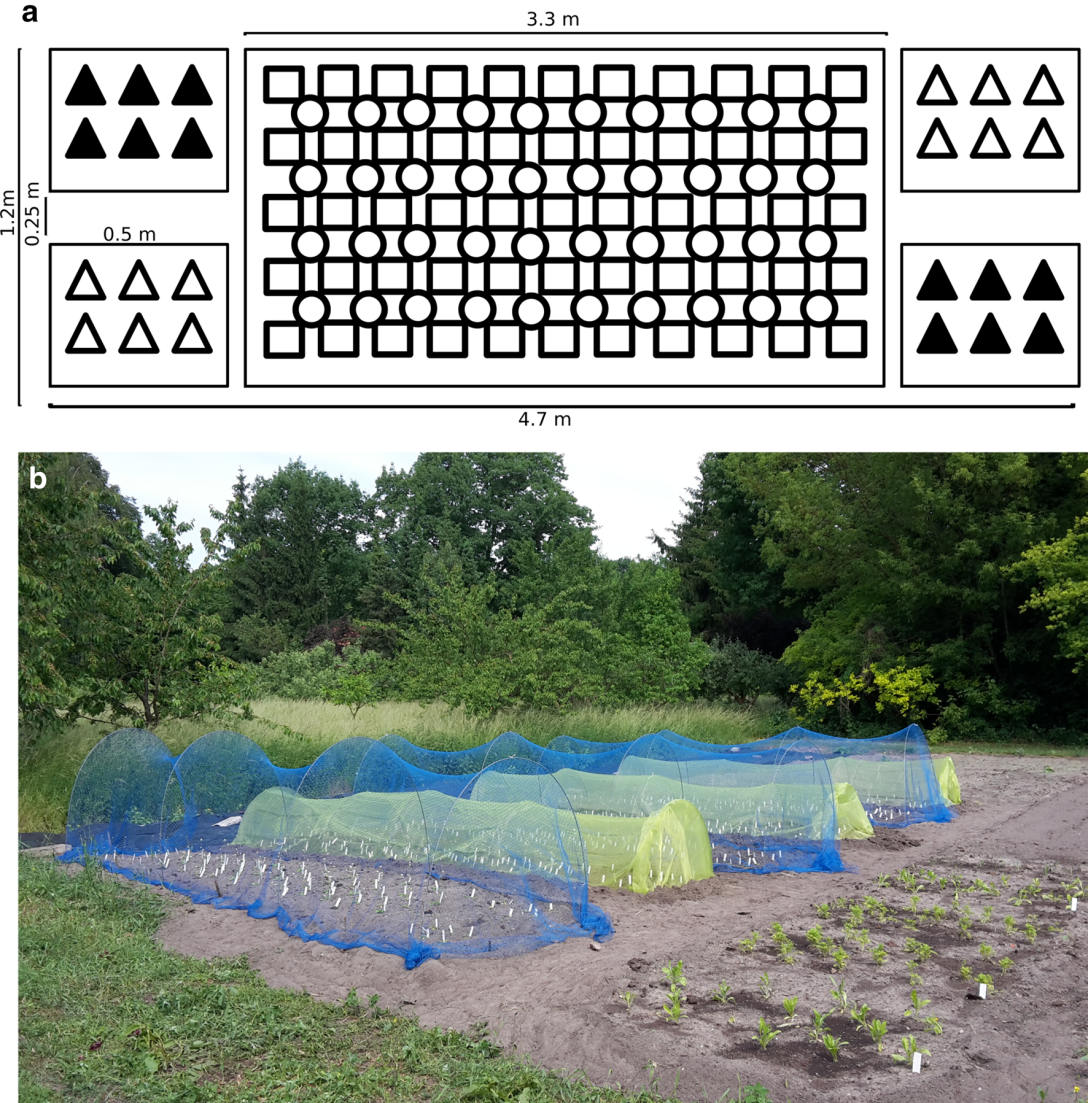
Plot set-up for common garden experiment. **a** Detailed experimental design. Every plot consisted of five patches: two with six scented qIL plants (black triangles) and two with six unscented qIL plants (white triangles). The big area in the centre contained other *Capsella* lines to provide pollen for outcrossing (circles-*Capsella grandiflora* self-compatible line, squares—NILs differing in petal size). **b** Photo of the common garden experiment from Spring 2017. Each plot was set up like described in 1A. In three plots plants were grown under insect-exclusion (green nets) and in the other three plots insects had access but plants were protected from other wildlife like birds and deer (blue nets)

Two *Capsella* qILs only differing in about 12 kb around the locus for loss of benzaldehyde emission were compared here [23]. A self-compatible *Capsella grandiflora* line and qILs segregating for petal size were used as sources to provide pollen for outbreeding [17, 24]. At the beginning of April 2017, seeds were sown on a soil-compost mix and watered with GA-supplemented water (gibberellic acid stock: 16.5 mg in 1 ml ethanol, 1:5000 dilution for watering) until germination. After sowing, seeds were stratified for 4 days in 4 °C and then transported into an open greenhouse. Seedlings were pricked out into individual pots when they showed 4–6 leaves at 3 weeks after germination and were planted out in the plots just before starting to flower in mid-May and bird or insect protection nets were installed. The set-up is shown in Fig. 1. Plants flowered during June and July and were bagged in July to be harvested in August. When the oldest fruits started to ripen, individual plants were bagged and allowed to ripen for two more weeks before collection and preparation for DNA extractions. Due to weather conditions and infection with herbivores low numbers of seeds were harvested from each plant. Seeds from all plants with the same genotype within one plot were pooled and cleaned. To be able to define parental haplotypes, we also collected leaf material from the two qILs and subjected it to the same analysis as the seed samples.

### DNA extraction from pooled seeds

This method was optimized for extracting DNA from *Capsella* seeds. Depending on seed number/size it may need to be adapted. Other extraction methods such as CTAB [25] did not give any results.

1. Approximately 300 seeds were counted into a 2 ml Eppendorf tube to reduce contamination with sand and other dirt. Sterilized water was added and seeds were incubated for 2 days at 4 °C.
2. Transfer seeds into the mortar, remove water by pipetting. Wash 2–3 times with water to remove dirt if necessary.
3. Cool down mortar and pistil with liquid nitrogen and grind samples to a fine powder. Transfer the powder into a new 2 ml tube and add 800 µl Buffer AP1 of Quiagen DNeasy Plant Mini Kit. Store on ice until all samples are ground.
4. Proceed with following steps from Quiagen Kit manual, add double amount of buffer AP2 in step 3. For elution in step 12 only 50 µl buffer AE was used.
5. Test extracted DNA in a PCR. If there is no amplification and starting material contained a lot of soil or organic matter, try cleaning DNA samples with e.g. AMPure beads.
6. Transfer DNA samples into 96-well plate for further steps.

### Design of PCR primers

Next-generation sequencing libraries relying on PCR-based approaches are known to be prone to numerous biases linked to amplicon length and GC-content, sequence heterogeneity at primer-annealing sites as well as copy number variation [26–29]. To limit amplification biases, we focused our analysis on low polymorphic genes present in a single copy within genomes. To this end, we retrieved sequences from single-copy nuclear genes from *C. rubella* reference genome (https://phytozome.jgi.doe.gov/pz/portal.html) based on [30]. Conserved sites within the *Capsella* genus were identified by comparing the sequence of 50 *C. rubella* and 193 *C. grandiflora* genomes. Primers were designed using Primer 3 plus in order to amplify amplicons of approximately 300 bp and to anneal to their templates at 55 °C. We added 33 and 34 nt sequences complementary to the forward and reverse index primers to each of the gene-specific forward and reverse primers, respectively.

Sequencing quality will also depend on the heterogeneity of sequences as it is used during the first amplification cycles for cluster identification and phasing/pre-phasing calibration [31]. Low-sequence heterogeneity may impair the distinction between different clusters and considerably limit the output of the sequencing run. A common solution to such issues is to co-sequence amplicon libraries with a heterogeneous control library (e.g. usually prepared from the bacteriophage PhiX genome and mixed at variable proportions, between 15 and 50%) with the drawback that a large number of reads will be lost as they will not correspond to the sequence of the target locus. Here, we introduce sequence heterogeneity by designing an additional primer pair for each target loci containing an additional nucleotide between the gene-specific sequence and those complementary to the indexing primers (Tables 1, 2).

**Table 1:** Primers for Primary PCR

Primer name	Sequence
1_Carubv10013869m_F	TCGTCGGCAGCGTCAGATGTGTATAAGAGACAGCCACTCATCCATTCGGAAAT
1_Carubv10013869m_R	GTCTCGTGGGCTCGGAGATGTGTATAAGAGACAGTTGGGGACAAGGTGCTAATC
2_Carubv10023806m_F	TCGTCGGCAGCGTCAGATGTGTATAAGAGACAGTACCGACCACATAGGCATCA
2_Carubv10023806m_R	GTCTCGTGGGCTCGGAGATGTGTATAAGAGACAGAATGGCCGATTCTGCTTTTA
3_Carubv10018138m_F	TCGTCGGCAGCGTCAGATGTGTATAAGAGACAGCAAGCCAAAGTTTGATGCTT
3_Carubv10018138m_R	GTCTCGTGGGCTCGGAGATGTGTATAAGAGACAGACTCGTCTGCAGTCATGGTG
4_Carubv10001640m_F	TCGTCGGCAGCGTCAGATGTGTATAAGAGACAGGGAAGCGGATGGTTACAAAA
4_Carubv10001640m_R	GTCTCGTGGGCTCGGAGATGTGTATAAGAGACAGAGGCCAAGCTCACTCACATT
5_Carubv10001924m_F	TCGTCGGCAGCGTCAGATGTGTATAAGAGACAGTGGGTTCAGATTGAGCGTAA
5_Carubv10001924m_R	GTCTCGTGGGCTCGGAGATGTGTATAAGAGACAGAACTTGATCCTCTTTGGTACTGG
6_Carubv10023818m_F	TCGTCGGCAGCGTCAGATGTGTATAAGAGACAGTTCTTTTTCTGAGATTCCATTGCT
6_Carubv10023818m_R	GTCTCGTGGGCTCGGAGATGTGTATAAGAGACAGAGAAGCCTCTCCTGAGAAGTGA
7_Carubv10005658m_F	TCGTCGGCAGCGTCAGATGTGTATAAGAGACAGTCCAAGATCTGTGCTTGCTG
7_Carubv10005658m_R	GTCTCGTGGGCTCGGAGATGTGTATAAGAGACAGTCAGCTCCGGATGGTTAAAT
8_Carubv10006001 m _F	TCGTCGGCAGCGTCAGATGTGTATAAGAGACAGTTTCAAAAGCTTTGCGTGAG
8_Carubv10006001 m_R	GTCTCGTGGGCTCGGAGATGTGTATAAGAGACAGGATGCTTCACGTTCACACCA
9_Carubv10006101m_F	TCGTCGGCAGCGTCAGATGTGTATAAGAGACAGGTTCTATCCAAGGGCCATCA
9_Carubv10006101m_R	GTCTCGTGGGCTCGGAGATGTGTATAAGAGACAGCCCATGGAAACTCCTTGTTG
10_Carubv10027375m_F	TCGTCGGCAGCGTCAGATGTGTATAAGAGACAGGATCCGTCGGCTCTTCTCTC
10_Carubv10027375m_R	GTCTCGTGGGCTCGGAGATGTGTATAAGAGACAGAACCATGCCAATGCTTCATA
11_Carubv10011729m_F	TCGTCGGCAGCGTCAGATGTGTATAAGAGACAGGGAGCAAGTCCCAAACAAAG
11_Carubv10011729m_R	GTCTCGTGGGCTCGGAGATGTGTATAAGAGACAGCATTTCAAGCCGCTCTGG
12_Carubv10014733m_F	TCGTCGGCAGCGTCAGATGTGTATAAGAGACAGTGCATTCGATCTCGATCTTG
12_Carubv10014733m_R	GTCTCGTGGGCTCGGAGATGTGTATAAGAGACAGCGGTGGTGAAGACAACAATC
*Primers targeting same sequences as above, but with added T or A before gene*-*specific sequence for heterogeneity*	
1A_Carubv10013869m_F	TCGTCGGCAGCGTCAGATGTGTATAAGAGACAGACCACTCATCCATTCGGAAAT
1A_Carubv10013869m_R	GTCTCGTGGGCTCGGAGATGTGTATAAGAGACAGATTGGGGACAAGGTGCTAATC
2T_Carubv10023806m_F	TCGTCGGCAGCGTCAGATGTGTATAAGAGACAGTTACCGACCACATAGGCATCA
2T_Carubv10023806m_R	GTCTCGTGGGCTCGGAGATGTGTATAAGAGACAGTAATGGCCGATTCTGCTTTTA
3A_Carubv10018138m_F	TCGTCGGCAGCGTCAGATGTGTATAAGAGACAGACAAGCCAAAGTTTGATGCTT
3A_Carubv10018138m_R	GTCTCGTGGGCTCGGAGATGTGTATAAGAGACAGAACTCGTCTGCAGTCATGGTG
4T_Carubv10001640m_F	TCGTCGGCAGCGTCAGATGTGTATAAGAGACAGTGGAAGCGGATGGTTACAAAA
4T_Carubv10001640m_R	GTCTCGTGGGCTCGGAGATGTGTATAAGAGACAGTAGGCCAAGCTCACTCACATT
5A_Carubv10001924m_F	TCGTCGGCAGCGTCAGATGTGTATAAGAGACAGATGGGTTCAGATTGAGCGTAA
5A_Carubv10001924m_R	GTCTCGTGGGCTCGGAGATGTGTATAAGAGACAGAAACTTGATCCTCTTTGGTACTGG
6T_Carubv10023818m_F	TCGTCGGCAGCGTCAGATGTGTATAAGAGACAGTTTCTTTTTCTGAGATTCCATTGCT
6T_Carubv10023818m_R	GTCTCGTGGGCTCGGAGATGTGTATAAGAGACAGTAGAAGCCTCTCCTGAGAAGTGA
7A_Carubv10005658m_F	TCGTCGGCAGCGTCAGATGTGTATAAGAGACAGATCCAAGATCTGTGCTTGCTG
7A_Carubv10005658m_R	GTCTCGTGGGCTCGGAGATGTGTATAAGAGACAGATCAGCTCCGGATGGTTAAAT
8T_Carubv10006001m _F	TCGTCGGCAGCGTCAGATGTGTATAAGAGACAGTTTTCAAAAGCTTTGCGTGAG
8T_Carubv10006001m_R	GTCTCGTGGGCTCGGAGATGTGTATAAGAGACAGTGATGCTTCACGTTCACACCA
9A_Carubv10006101m_F	TCGTCGGCAGCGTCAGATGTGTATAAGAGACAGAGTTCTATCCAAGGGCCATCA
9A_Carubv10006101m_R	GTCTCGTGGGCTCGGAGATGTGTATAAGAGACAGACCCATGGAAACTCCTTGTTG
11T_Carubv10011729m_F	TCGTCGGCAGCGTCAGATGTGTATAAGAGACAGTGGAGCAAGTCCCAAACAAAG
11T_Carubv10011729m_R	GTCTCGTGGGCTCGGAGATGTGTATAAGAGACAGTCATTTCAAGCCGCTCTGG
12A_Carubv10014733m_F	TCGTCGGCAGCGTCAGATGTGTATAAGAGACAGATGCATTCGATCTCGATCTTG
12A_Carubv10014733m_R	GTCTCGTGGGCTCGGAGATGTGTATAAGAGACAGACGGTGGTGAAGACAACAATC

**Table 2:** Primers for indexing PCR (indexed sequencing primers)

Primer name	Sequence	Index set origin	index name
F1_MetaIndex	AATGATACGGCGACCACCGAGATCTACACTATAGCCTTCGTCGGCAGCGTC	TruSeq i5	D501
F2_MetaIndex	AATGATACGGCGACCACCGAGATCTACACATAGAGGCTCGTCGGCAGCGTC	TruSeq i5	D502
F3_MetaIndex	AATGATACGGCGACCACCGAGATCTACACCCTATCCTTCGTCGGCAGCGTC	TruSeq i5	D503
F4_MetaIndex	AATGATACGGCGACCACCGAGATCTACACGGCTCTGATCGTCGGCAGCGTC	TruSeq i5	D504
F5_MetaIndex	AATGATACGGCGACCACCGAGATCTACACAGGCGAAGTCGTCGGCAGCGTC	TruSeq i5	D505
F6_MetaIndex	AATGATACGGCGACCACCGAGATCTACACTAATCTTATCGTCGGCAGCGTC	TruSeq i5	D506
F7_MetaIndex	AATGATACGGCGACCACCGAGATCTACACCAGGACGTTCGTCGGCAGCGTC	TruSeq i5	D507
F8_MetaIndex	AATGATACGGCGACCACCGAGATCTACACGTACTGACTCGTCGGCAGCGTC	TruSeq i5	D508
R13_MetaIndex	CAAGCAGAAGACGGCATACGAGATGTCGTGATGTCTCGTGGGCTCGG	TruSeq Amplicon	A701
R14_MetaIndex	CAAGCAGAAGACGGCATACGAGATCGAGTAATGTCTCGTGGGCTCGG	TruSeq i7	D701
R15_MetaIndex	CAAGCAGAAGACGGCATACGAGATTCTCCGGAGTCTCGTGGGCTCGG	TruSeq i7	D702
R16_MetaIndex	CAAGCAGAAGACGGCATACGAGATAATGAGCGGTCTCGTGGGCTCGG	TruSeq i7	D703
R17_MetaIndex	CAAGCAGAAGACGGCATACGAGATGGAATCTCGTCTCGTGGGCTCGG	TruSeq i7	D704

### PCR amplification and library generation

The method for PCR amplification and library preparation is based on a recent protocol for amplicon-based microbiome characterization [32]. Further details and troubleshooting information can be found in [32].

This protocol is optimized for low sample DNA concentrations due to the availability and quality of starting material. For best results, minimize PCR cycles and select the samples with the highest concentrations in step 1c. Sample dilution might result in bottlenecking for low abundance alleles as described [32].

**Fig. 2:**
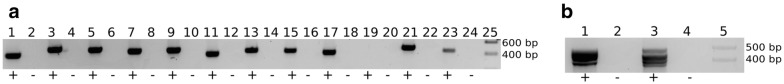
Primer-test for individual primers and pooling strategy. **a** Primer sets 1–12 were tested with genomic DNA as template (odd-numbered lanes, ‘ + ’) or in a no-template control reaction (even-numbered lanes, ‘ − ’). Primer set 10 (lanes 19 and 20) gave no PCR product and was therefore excluded in further steps. **b** Pooled amplification of primer sets 1–5 (lanes 1 and 2) and 6–12 (without 10, lane 3 and 4) with water controls
    Thaw the Kapa HiFi Hotstart kit reagents and the 384-sample plate if it was stored in the freezer. Vortex and centrifuge all reagents when thawed before using.
    Prepare a 2 × KAPA HiFi Hotstart qPCR master mix with the following components: 1.2 µl 5 × KAPA HiFi Fidelity buffer, 0.18 µl 10 mM dNTPs, 0.3 µl DMSO, 0.12 µl ROX (25 µM), 0.003 µl 1000 × SYBR Green, 0.12 µl KAPA HiFi Hotstart Polymerase, 0.3 µl forward primer pool (10 µM), 0.3 µl reverse primer pool (10 µM) and 0.48 µl nuclease-free water.
    Dispense 3 µl of 2 ×  KAPA HiFi Hotstart qPCR master mix in each reaction well on the 384-well plate containing DNA samples for a final volume of 6 µl.
    Cover the plate, mix and spin-down. Start the following qPCR protocol on Roche LightCycler 480 II (or similar) after loading the plate: 95 °C for 5 min, then 15 cycles of 98 °C 20s, 55 °C for 15s, 72 °C for 1 min. Cycle number can be between 15 and 30 cycles but optimal results will be achieved by keeping the cycle number low. However, samples amplifying poorly could be amplified using more cycles. Plates can be stored at -20 °C.
  - Analysis and choosing the best dilution for indexing PCR
    The analysis was done manually. For more details on how to conduct it automatically, please refer to [32].
    Compare the amplification curves for the different dilutions of the same DNA sample. Choose a sample concentration which is in the mid-to-late exponential phase at the last amplification cycle of the PCR for further steps. Samples should not have reached a plateau at the final cycle as this means overamplification. Figure 3a, b show two examples of amplification curves, due to low DNA concentrations samples were only in the early-exponential phase but were still used for further steps.

1. Primary PCR
  - Create a sample dilution plate.
    Vortex DNA samples and spin down in a centrifuge. Prepare a 384-well plate by pipetting 18 µl water in quadrant 2 (A02), 3 (B01) and 4 (B02). Dispense 10 µl of the undiluted sample into quadrant 1 (A01). Generate a tenfold dilution series by transferring 2 µl of each sample to quadrant 2. Pipet up and down ten times for mixing. Repeat for quadrants 3 and 4. In the end, you will have your undiluted sample in quadrant 1, 1:10 dilution in quadrant 2, 1:100 dilution in quadrant 3 and 1:1000 dilution in quadrant 4.
    For primary PCR, pipet 3 µl of every sample and dilutions into a new 384-well plate which can be used in LightCycler 480 II. Start with the lowest concentration in quadrant 4 (B02) and then proceed with quadrant 3, 2 and 1 to use the same set of tips. Plates can be stored at − 20 °C or used for subsequent primary PCR.
  - Primary PCR
    Prior to PCR, test primers individually and pooled for amplification of target genes. Figure 2a shows amplification products for 11 out of 12 primer pairs, in Fig. 2b the pooling strategy was tested for two sets of primer pairs. For the primary PCR, a maximum of six different primer pairs was pooled (including primer pairs with added bases for heterogeneity, so 12 primer pairs overall).

**Fig. 3:**
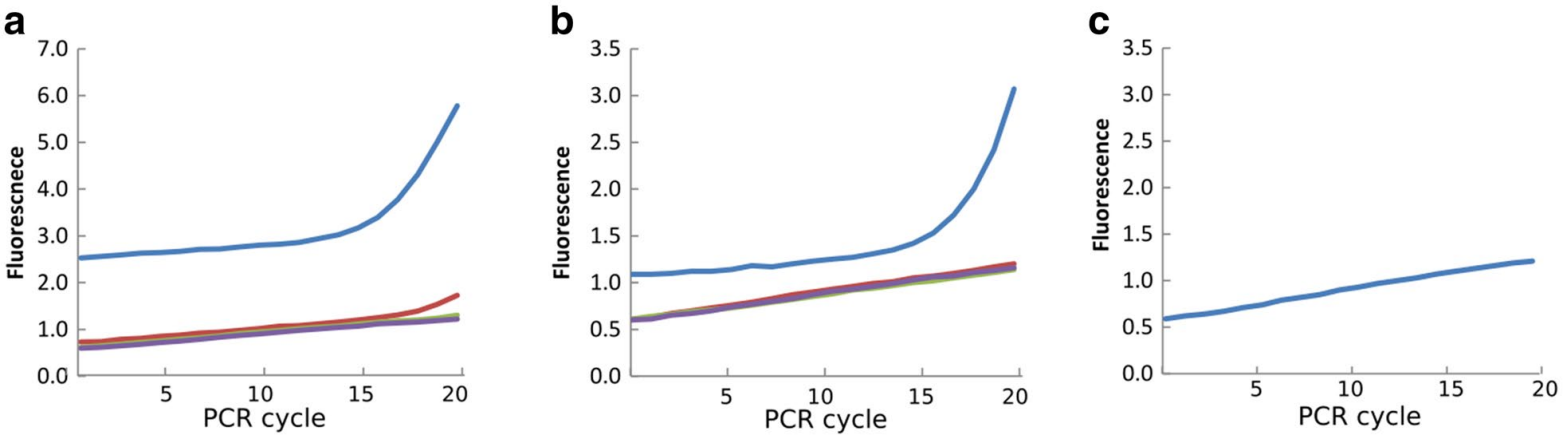
Examples for qPCR amplification traces. For **a**, **b** blue trace is the undiluted sample, red trace is 1:10 dilution, green is 1:100 and purple trace is 1:1000 dilution. DNA concentrations were low enough to not overamplify within 20 cycles. For more detailed information about cherry-picking using a robot please refer to the protocol provided by Gohl and colleagues [32]. **a** Undiluted sample (blue) is in mid-exponential phase and was used for further steps. 1:10 dilution is in early-exponential phase, the two lowest dilutions show no amplification. **b** Undiluted sample (blue) is in early-to-mid-exponential phase and was used for further steps. Other dilutions did not amplify. **c** Water blank control
    Prepare 96 well plate by distributing 18 µl water in each well. Spin down qPCR plate and transfer 2 µl of the appropriate dilution into the new plate to create a 1:10 dilution of the primary PCR. Mix and spin down. (If samples were in mid-to-late exponential phase after the final PCR cycle, transfer 5 µl of 1:10 dilution into a new plate containing 45 µl water to generate a 1:100 dilution of primary PCR. Use this instead of 1:10 dilution for further steps). Store at − 20 °C or progress to indexing PCR.
2. Indexing PCR
  - Picking an indexing scheme
    Each sample needs to have an individual combination of i5 and i7 indices to make sure no index overlap between pooled samples can occur. Prior to running the PCR an i5 and i7 dual-indexing scheme needs to be chosen, depending on how many samples will be pooled together for sequencing. The number of samples is equal to the number of index combinations needed.
    See Illumina guide for more information on dual indexing: https://support.illumina.com/content/dam/illumina-support/documents/documentation/system_documentation/miseq/indexed-sequencing-overview-guide-15057455-04.pdf
    For the design of Illumina adapter sequences see: https://support.illumina.com/content/dam/illumina-support/documents/documentation/chemistry_documentation/experiment-design/illumina-adapter-sequences-1000000002694-09.pdf
  - Indexing PCR
    Prepare an oligo plate, adapted to your indexing scheme.
    Make 10 µM dilutions of your 100 µM primer stocks in a 96 well plate by adding 30 µl 100 µM oligo stock to 270 µl water; forward primers were arranged in columns and reverse primers in rows.
    For the 5 µM master plate containing 40 µl primer mix, add 10 µl of the 10 µM forward and reverse primer dilutions into a new plate and add 20 µl water. Mix well and use 2 µl for indexing PCR. See Additional file 1: Table S1 for indexing scheme used in this study. [Please note that of the 38 different index combinations shown in Additional file 1: Table S1, only 12 were used for samples analyzed in this study.]
    Thaw the KAPA HiFi Hotstart PCR kit reagents and the primary PCR dilution plate (1:10 or 1:100) if necessary. Mix and spin down reagents.
    Prepare a 3.33 KAPA HiFi Hotstart Indexing PCR master mix consisting of these components: 2 µl 5 × KAPA HiFi Fidelity buffer, 0.3 µl 10 mM dNTPs, 0.5 µl DMSO, 0.2 µl KAPA HiFi Hotstart Polymerase. Dispense 3 µl into wells of a 96-well PCR plate. Add 5 µl of diluted primary PCR product to the corresponding wells in the 96-well PCR plate that contains 3 µl of the indexing PCR master mix. Add 2 µl of 5 µM indexing primers from the prepared oligo plate for indexing. The final reaction volume is 10 µl.
    Seal the plate, mix and spin down. Amplify in PCR-machine with the following conditions: 95 °C for 5 min, 10 cycles of 98 °C for 20 s, 55 °C for 15 s, 72 °C for 1 min. Centrifuge the plate to collect the samples after PCR program is complete. The plate can be stored at − 20 °C.
3. Normalization and pooling
  There are different options for normalization and pooling, depending on available systems and reagents. Please check protocol by Gohl et al. for further information [32].
  - QuantiFluor quantification of indexed samples
    Calculate DNA concentrations of indexed samples following the manufacturer’s protocol for the QuantiFluor dsDNA system. Use two times 1 µl indexing PCR reaction per sample for quantification, so 8 µl are left for normalization.
  - Sample normalization
    Choose a concentration for normalization. It depends on how concentrated/diluted your samples are. Calculate the amount of water for each sample to be added to a fixed amount of indexing PCR in order to get the desired concentration. For this experiment, 4 µl indexing PCR were used and 5 ng/µl were determined as desired concentration. The amount of water to add fluctuated from 0 to 90 µl, depending on sample concentration.
    Seal plate, mix and spin down. The plate can be stored at − 20 °C.
  - Pooling and clean-up
    Pool identical volumes of all samples for the library. Here, 3.5 µl of each sample normalized to 5 ng/µl were used. Mix and transfer to a 1.5 ml Eppendorf tube. Use Speedvac to collect pool in 20–100 µl. Clean sample pool by using 1 ×  AMPure XP beads (see Appendix 2 of [32]) and elute in 20 µl TE buffer.
  - Verify quality and size distribution of pooled library
    The expected size distribution should be around 500 bp, as primers for primary PCR were designed to keep variable regions between 300 and 400 bp long and approximately 170 bp were added for Illumina Sequencing (including sequences for read 1 and read 2, indices and i5/i7). Verify size range and distribution by running the library on 2200 TapeStation, following the manufacturer’s protocol.
    Determine library concentration by using Qubit 2.0 Fluorometric Quantification and following the manufacturer’s protocol.
    Submit the library to your sequencing facility or follow instructions provided by [32].

### Amplicon sequencing

**Fig. 4:**
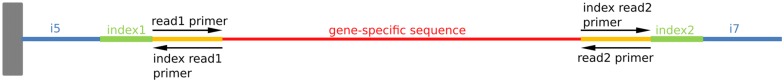
Structure of the final amplicon. Gene-specific sequences are amplified during primary PCR (red), read1 and read 2 sequences (orange) are added to the gene-specific primers. Indices (green) and i5/i7 sequences (blue) are added during indexing PCR

Sequencing can be performed on a NextSeq sequencing platform using a NextSeq Reagent Kit MidOutput (300 cycles). The library is compatible with the standard sequencing primers, final amplicon structure is shown in Fig. 4.

### Sequence analysis and estimation of outcrossing rates

Read pairs were associated with amplicons by parsing for the presence of forward and reverse primer at the beginning of reads 1 and 2 respectively using cutadapt version 2.1 [33]. Primer sequences were removed, including preceding T or A nucleotides that were added for heterogeneity (see Table 1). Reads resulting from primers without those T or A nucleotides were cut by one additional nucleotide at the end to get identical read lengths for both primer versions. Only reads corresponding to the expected length were kept: sequencing length—length of gene-specific sequence part of the primer—one nucleotide for T or A. Obtained sequences for read 1 and 2 were combined into one fragment to be treated as haplotype further on. Haplotype occurrences were counted. Haplotypes with a frequency below 1% were excluded from further analyses as they are likely to be a consequence of random sequencing errors; we expect this not to shift haplotype proportions. Haplotypes with a frequency above 1%, but only present in one single sample were also excluded as they are likely to be a consequence of an early PCR amplification error. Roughly half of the fragments were filtered out that way (see Additional file 2: Table S2). The sums of remaining fragments per sample and amplicon were then used as a baseline to calculate (1) the proportions of parental (P1 or P2) haplotypes per sample and amplicon and (2) the proportions of P1 and P2 haplotypes for the polymorphic amplicon 6_Carubv10023818 m. Data analyses were done using R (https://www.r-project.org). Illustrations were done using the R/lattice package (http://lmdvr.r-forge.r-project.org).

## Results

Plants of two *Capsella* qILs differing in about 12 kb around the *CNL1* locus that underlies the loss of benzaldehyde emission in *C. rubella* [23] were grown at the field site of the Botanical Garden of the University of Potsdam. The arrangement of the plants is shown in Fig. 1. Each block contained two sets of six plants each of both genotypes arranged on opposite sides of a central area with inbred, self-compatible *C. grandiflora* plants and near-isogenic lines differing for the *SAP* locus that affects petal size [24]. The plants in this central area served as pollen donors with different background genotypes. Three such blocks were protected from birds and rodents by a bird net, while three such blocks were covered by an insect-proof net.

We designed 12 primer pairs in exons of highly conserved genes that anneal to invariant nucleotide stretches across a large number of *C. grandiflora* and *C. rubella* genotypes. Primers were chosen in exons flanking an intron to maximize the sensitivity for detecting non-maternal genotypes, as intron sequences are generally more variable than exonic sequences. As shown in Fig. 2, 11 out of the 12 primer pairs successfully amplified and two pools of six and five primer pairs were set up to minimize the number of required PCR reactions.

Genomic DNA was extracted from approximately 300 pooled seeds from the 12 scented and the 12 non-scented qIL plants per block. A previously described qPCR-based approach [32] was used to determine the optimal template concentration for the primary PCRs with the two pools of six and five primer pairs described above. Example results for this test are shown in Fig. 3. In most cases, the undiluted sample was used for further steps.

**Fig. 5:**
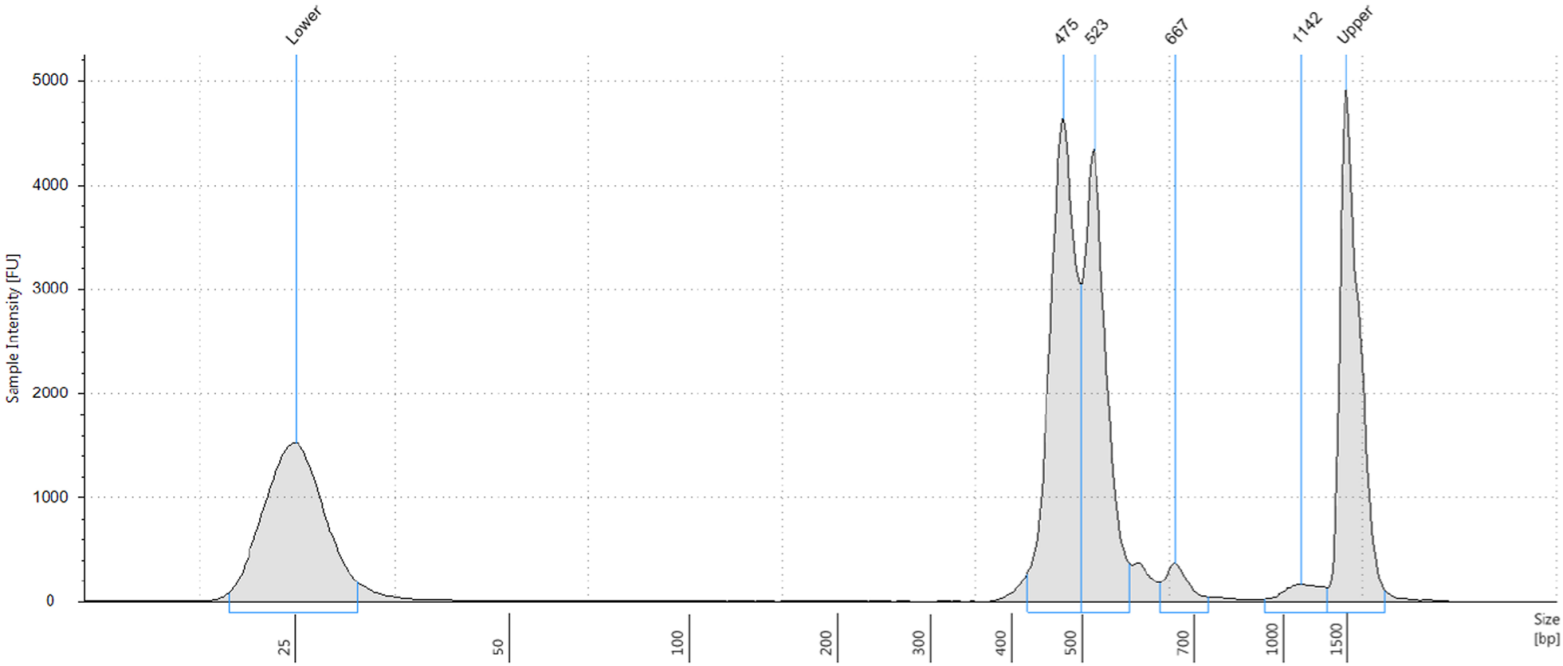
Example of a final library as analyzed on a Tape Station. The library fragments are between 400 and 600 bp long

Barcoding indices were introduced via a second, indexing PCR, resulting in final amplification products with the structure shown in Fig. 4. Products from the two primary PCRs (with the six- and five-primer pair pools, respectively) were combined in equimolar ratios after this indexing PCR. An example of a final library pool as determined by Tape Station electrophoresis is shown in Fig. 5.

**Fig. 6:**
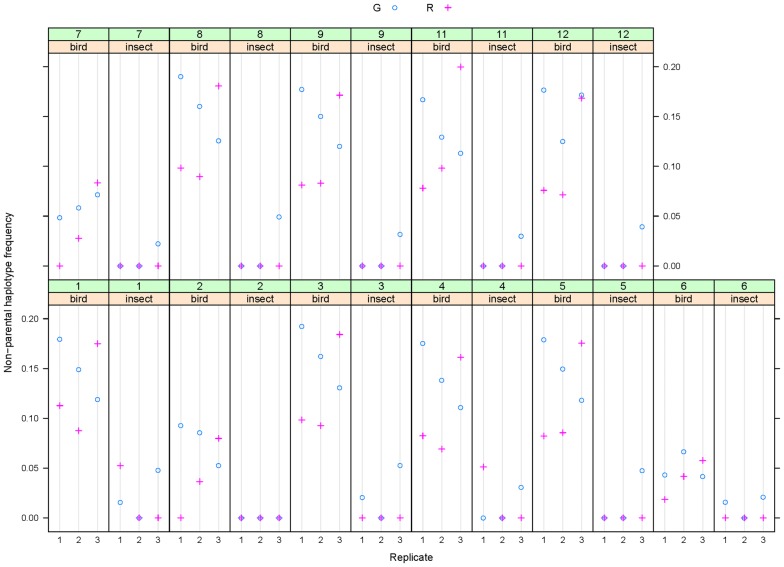
Non-parental haplotype frequencies across the amplicons. Non-parental haplotype frequencies are plotted across the 11 amplicons. Amplicons are indicated by numbers ‘1′ to ‘12’, according to Table 1. Paired results for each of the three replicated blocks under the bird nets (‘bird’) and insect nets (‘insect’) are shown, with replicates numbered 1 to 3. ‘G’ and ‘R’ indicate samples homozygous for the *C. grandiflora* allele or the *C. rubella* allele in the *CNL1* region, respectively. For amplicon 6, only those haplotypes were counted as non-parental that were distinct from those found in either of the parental lines. The results of statistical comparisons between the two genotypes under one type of net and between all samples under bird versus under insect nets are given in Additional file 3: Table S3

**Fig. 7:**
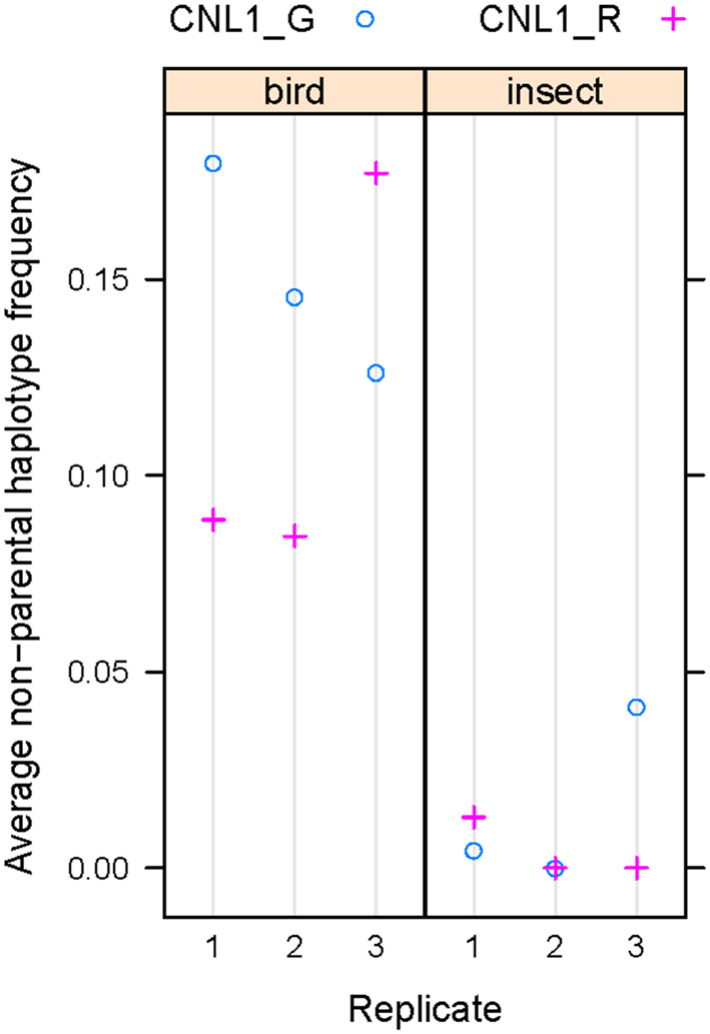
Mean non-parental haplotype frequencies across the high-scoring eight amplicons. Mean values across the amplicons 1, 3, 4, 5, 8, 9, 11, 12 are plotted for the twelve samples. Statistical analysis by paired *t* test between the two genotypes under bird nets or between the two genotypes under insect nets did not detect any significant difference (p > 0.05 in both cases). By contrast, the difference between all samples under bird nets versus all samples under insect nets was highly significant at p < 0.001 based on a Welch two-sample t-test

Libraries were sequenced in 2 × 150 bp paired-end mode, and the two reads were linked to create 300-bp fragments for analysis. Based on these, we defined major haplotypes as those present with at least 1% frequency in more than one sample. This excludes low-frequency sequencing errors, but also PCR-errors from early cycles, as these should be unique to individual samples. After such filtering, around 50% of fragments remained for analysis, except for sample 26, where only 35% of fragments were retained (Additional file 2: Table S2). We assigned these remaining fragments to either parental or non-parental haplotypes by comparison to the results from the leaf samples of the qILs processed in parallel. Across all amplicons, the frequency of non-parental reads was consistently higher in the samples from plants grown under the bird-protection nets only, i.e. accessible to insects, than from those grown under insect-proof nets (Fig. 6); in fact, in the latter no non-parental haplotypes could be detected for eight of the amplicons in five out of the six samples. In the samples from the bird nets, the frequency of non-parental haplotypes reached between 10 and 20% for eight of the amplicons, with very consistent estimates across the individual samples. For three of the amplicons, the frequency of non-parental haplotypes was below 10% in the bird-net samples, again with consistent estimates across the individual samples. We ascribe this difference between the two groups of amplicons to haplotype sharing with the other plants in the blocks that served as pollen donors for the outbred seeds, with at least some of the genotypes sharing the parental haplotypes with our lines at the three amplicons in question, thus rendering many outcrossing events undetectable. Given the simple genetic structure of the pollen-donor populations (two NILs and one inbred *C. grandiflora*-like line), the extent of haplotype sharing is most likely the same for the eight amplicons with the higher estimates. Thus, the average values across these eight amplicons (Fig. 7) represent the basis of our best estimate of the outcrossing rate in our samples; in particular, given the diploid nature of the plants, the frequency of non-parental haplotypes has to be multiplied by two to obtain an estimate for the fraction of outcrossed seeds, i.e. the outcrossing rate. These values are shown in Table 3. While these values were higher for the samples from plants with the *C. grandiflora CNL1* haplotype than with the *C. rubella* haplotype for two of the replicates under the bird net, this was reversed in the third replicate. Thus, overall there was no consistent difference in the estimated outcrossing rate between benzaldehyde-emitting and non-emitting plants in this one trial.

**Table 3:** Estimated outcrossing rates (based on Fig. 7)

Genotype	Net	Replicate	Outcrossing rate
CNL1_G	Bird	1	0.36
CNL1_G	Bird	2	0.29
CNL1_G	Bird	3	0.25
CNL1_R	Bird	1	0.18
CNL1_R	Bird	2	0.16
CNL1_R	Bird	3	0.35
CNL1_G	Insect	1	0.01
CNL1_G	Insect	2	0
CNL1_G	Insect	3	0.08
CNL1_R	Insect	1	0.03
CNL1_R	Insect	2	0
CNL1_R	Insect	3	0

In summary, the strong and consistent difference in apparent outcrossing frequencies between samples under the two types of nets strongly supports the validity of our analysis method and indicates a surprisingly high rate of insect-mediated outcrossing in these self-compatible *Capsella* genotypes.

**Fig. 8:**
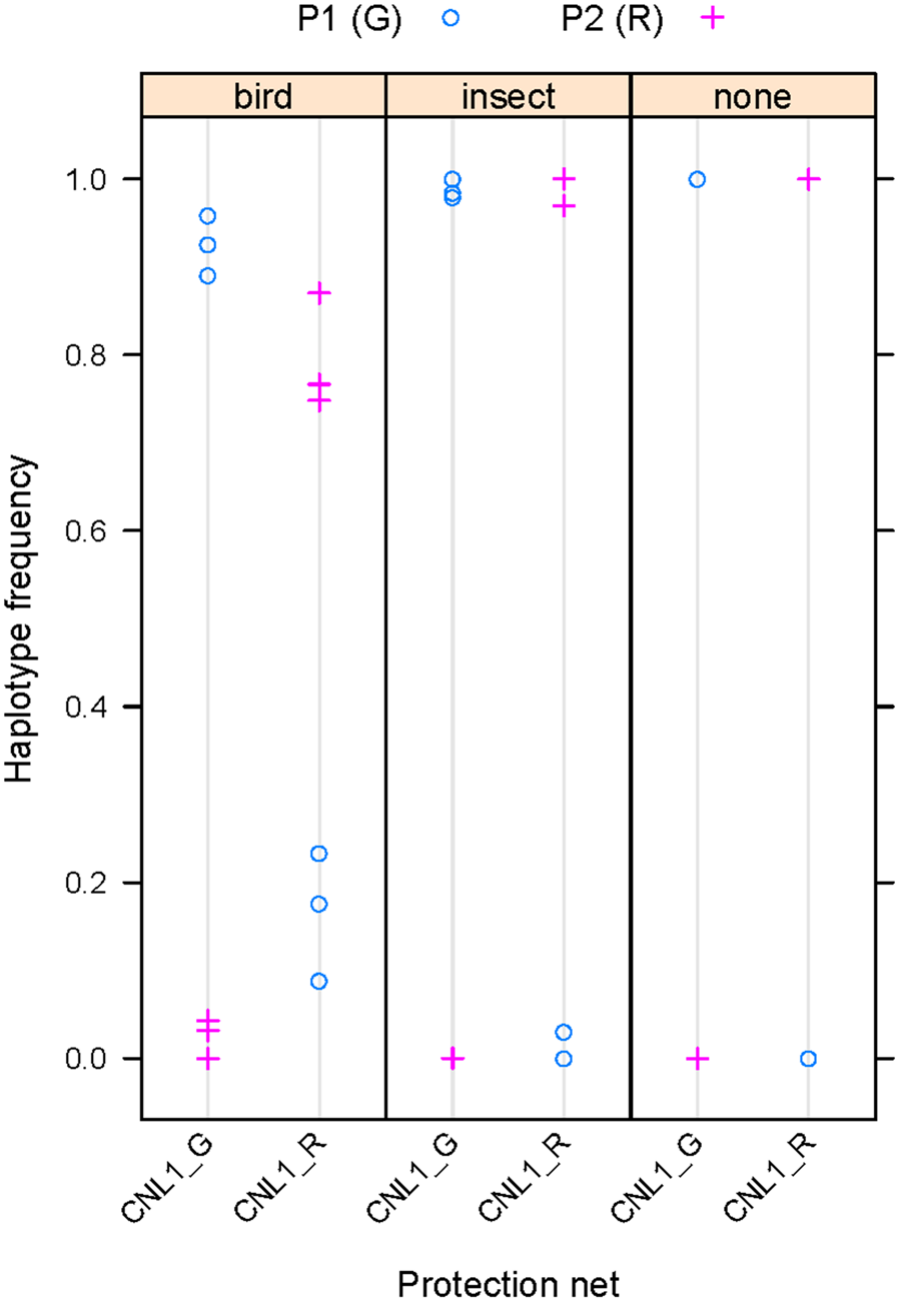
Frequency of the two alternative parental haplotypes in reads for amplicon 6 (Carubv10023818 m). Frequencies of the two alternative parental haplotypes at locus Carubv10023818 m (termed ‘P1 (G)’ and ‘P2 (R)’) is plotted for the samples carrying the *C. grandiflora* allele (CNL1_G) or the *C. rubella* allele (CNL1_R) in the *CNL1* region, respectively. Samples are separated according to the protection net they were under. ‘none’ indicates samples from selfed parental plants grown in the absence of animal pollinators. Note that only the two parental haplotypes were considered for this analysis. Statistical analysis by paired t-test between the two genotypes under bird nets or between the two genotypes under insect nets did not detect any significant difference. Similarly, comparison of all samples under bird nets with all samples under insect nets by a Welch two-sample t-test did not find a significant difference

We also found that the two parental lines differed at one of the amplicons (number 6, locus Carubv10023818 m), indicating that their genomic background was not fully isogenic. In principle, this difference could allow estimating outcrossing between the two parental lines. When considering only the two parental haplotypes, the plants with the *C. rubella* haplotype in the *CNL1* region appeared to have received more pollen with the alternative haplotype at amplicon 6 (i.e. from the plants with the *C. grandiflora* haplotype at *CNL1*) than vice versa under the bird nets (Fig. 8). While this difference could suggest asymmetric pollen flow between the two lines, it could also be due to differential haplotype sharing with the other plants in the plots, in particular, if the plants carrying the *C. grandiflora CNL1* haplotype shared their amplicon-6 haplotype with more of the other pollen-donor plants. To circumvent this issue of differential haplotype-sharing, only haplotypes found in neither of the two parental lines were counted for the analysis at amplicon 6 shown in Fig. 6.

## Discussion

In this study, we have described a method for estimating outcrossing rates that lends itself to the analysis of many samples with high throughput and have validated the method using a common-garden experiment comparing the outcrossing rate between open-pollinated and insect-excluded plants. Our results clearly show that the method successfully detects insect-mediated outcrossing events and provides consistent estimates of outcrossing rates across replicated samples. While the analysis approach presented assumes that the maternal genotypes at the tested amplicons are known, the method can easily be adapted to the case when these are not, by preparing a parallel set of amplicon-sequencing libraries from genomic DNA of the mother plants to be analyzed.

The described method offers a major advantage regarding the time and effort required to estimate the outcrossing rate for many samples. For example, obtaining the estimates for the twelve samples in our study using the classical method would have required running more than 10,000 individual PCR reactions and analyzing the products by electrophoresis (assuming 100 progeny seeds were genotyped per sample). At the same time, the degree of pooling libraries derived from different samples for the sequencing run could easily be increased, enabling the analysis of more samples with very little extra effort. For example, when demanding on average a 10,000-fold coverage for each of ten amplicons per sample, hundreds of samples could be analysed in parallel using a single NextSeq mid-output run. In principle, this would allow very fine-scale descriptions of how the outcrossing rate differs across a population to determine the effect of environmental influences or trait variation.

Compared with the single progeny-based approach these advantages concerning throughput come at a cost regarding the up-front investment in primer design and the precision of the estimates of outcrossing rates. As for primer design, one obvious technical source of error is differential amplification efficiency of different haplotypes due to mismatches in the primer-binding sites. For individual-based measurements, only very large differences in amplification efficiencies will cause an error, causing, for example, certain heterozygotes to be called as homozygotes for the more efficiently amplifying allele. By contrast, even slight amplification biases for different haplotypes can cause substantial error in the estimated outcrossing rates using the method described here. To circumvent this issue, some up-front sequencing of the haplotypes in the population in question may be necessary to identify highly conserved primer-binding sites. Following the strategy taken here, a cost-effective method for doing so would be to sequence PCR amplification products from highly conserved genes containing one or more introns from many pooled individuals in the population. Analysis of these sequences should allow identifying invariant primer-binding sites flanking suitably polymorphic regions or adapting the primer design by incorporating polymorphic bases into the primers, if no fully invariant regions can be found. The strategy for primer design is outlined in Additional file 4: Figure S1.

As for the precision of the estimates, the present method will necessarily underestimate the true outcrossing rates, and it will do so more strongly than the individual-based method in most cases. Concerning each single amplicon, the true outcrossing rate will be underestimated by the combined frequency of the one or two maternal alleles in the pollen population, as any outcrossing event involving a pollen-carrying one of the maternal alleles will be undetectable when considering a single amplicon. The individual-based method is better able to deal with this complication than the pool-based approach. This is because for the individual-based method a single marker with a non-maternal allele or haplotype is enough to classify an individual as resulting from outbreeding. By contrast, this information is necessarily lost in our pool-based approach; in a hypothetical example, if there were ten such outbred individuals, each with the diagnostic non-maternal haplotype in a different amplicon, these would all be detectable in the individual-based approach, but would only be counted as a single outbreeding event in the pool-based approach. Such a scenario of maternal haplotype-sharing at many of the amplicons will result for example from bi-parental inbreeding [13, 14].

The above bias means that the estimate closest to the true outcrossing rate will be obtained from the amplicon for which the combined frequency of the maternal haplotypes in the pollen population is lowest. In this regard, longer sequence reads appear preferable, as they will allow detecting a larger number of different haplotypes in the population, thus reducing the described effect. A further implication of the above is that outbreeding rates are strictly only comparable between individuals carrying the same maternal haplotypes at a given amplicon, as these will be affected by the above bias in the same manner. This is exemplified by amplicon 6 in our study, for which the two parental lines were polymorphic. Here, merely counting non-maternal haplotypes would have given very different estimates for the two parental lines for this amplicon. Thus, in light of these issues, if the aim is to characterize the reproductive system in a population with unknown genetic structure in great detail, considering also aspects like bi-parental inbreeding, the individual-based method remains the method of choice. By contrast, the pool-based method described here should be preferable, if the main aim is to obtain relative outcrossing rates from a large number of individuals and in situations where the above biases are likely to have a weak effect.

In summary, we have described a cost-effective method for the high-throughput estimation of outcrossing rates in plants. We see its major application in studies to correlate outcrossing rates with environmental, morphological or physiological parameters across a large number of individuals, especially if the genetic structure of the population in question is known. This should enable connecting genetic differences that affect pollinator-attraction traits with effects on pollinator behaviour in ecologically realistic settings.

## Additional files

**Additional file 1: Table S1.** Example indexing scheme. An example indexing scheme is shown in a 96-well format. Please refer to Table 2 for sequences of the indices.

**Additional file 2: Table S2.** Read statistics for the amplicons (see attached Excel sheet). The total number of fragments (i.e. paired reads) per sample is shown, as is the number and percentage of fragments mapped to the PCR amplicons, and the number and percentage of fragments used for haplotype calling. The latter excluded low-frequency fragments (< 1%), as these most likely represent PCR or sequencing errors.

**Additional file 3: Table S3.** Results of statistical comparisons for the data shown in Fig. 6.

**Additional file 4: Figure S1.** Strategy for primer design. An outline for choosing suitable primer binding sites and designing primers is shown for different scenarios, along with suggested parameter values for the primers and amplicons.

## Data Availability

The datasets generated and analysed during the current study are available in the NCBI SRA repository under accession number PRJNA529581 (https://www.ncbi.nlm.nih.gov/sra/PRJNA529581).
