# Supplementary material for: A high-throughput amplicon-based method for estimating outcrossing rates
Source: Plant Methods. 2019 May 18;15:47. doi: 10.1186/s13007-019-0433-9 (PMC6525360; doi:10.1186/s13007-019-0433-9)
Supplement: Supplementary file 1 — Additional file 1: Table S1. Example indexing scheme. An example indexing scheme is shown in a 96-well format. Please refer to Table 2 for sequences of the indices. [file 13007_2019_433_MOESM1_ESM.docx]

**Table S1: Example indexing scheme.**

|  | 1 | 2 | 3 | 4 | 5 | 6 | 7 | 8 | 9 | 10 | 11 | 12 |
| --- | --- | --- | --- | --- | --- | --- | --- | --- | --- | --- | --- | --- |
| A | F1+R13 | F1+R14 | F1+R15 | F1+R16 | F1+R17 |  |  |  |  |  |  |  |
| B | F2+R13 | F2+R14 | F2+R15 | F2+R16 | F2+R17 |  |  |  |  |  |  |  |
| C | F3+R13 | F3+R14 | F3+R15 | F3+R16 | F3+R17 |  |  |  |  |  |  |  |
| D | F4+R13 | F4+R14 | F4+R15 | F4+R16 | F4+R17 |  |  |  |  |  |  |  |
| E | F5+R13 | F5+R14 | F5+R15 | F5+R16 | F5+R17 |  |  |  |  |  |  |  |
| F | F6+R13 | F6+R14 | F6+R15 | F6+R16 | F6+R17 |  |  |  |  |  |  |  |
| G | F7+R13 | F7+R14 | F7+R15 | F7+R16 |  |  |  |  |  |  |  |  |
| H | F8+R13 | F8+R14 | F8+R15 | F8+R16 |  |  |  |  |  |  |  |  |

An example indexing scheme is shown in a 96-well format. Please refer to Table 2 for sequences of the indices.
