# Supplementary figures and images for: A high-throughput amplicon-based method for estimating outcrossing rates
Source: Plant Methods. 2019 May 18;15:47. doi: 10.1186/s13007-019-0433-9 (PMC6525360; doi:10.1186/s13007-019-0433-9)

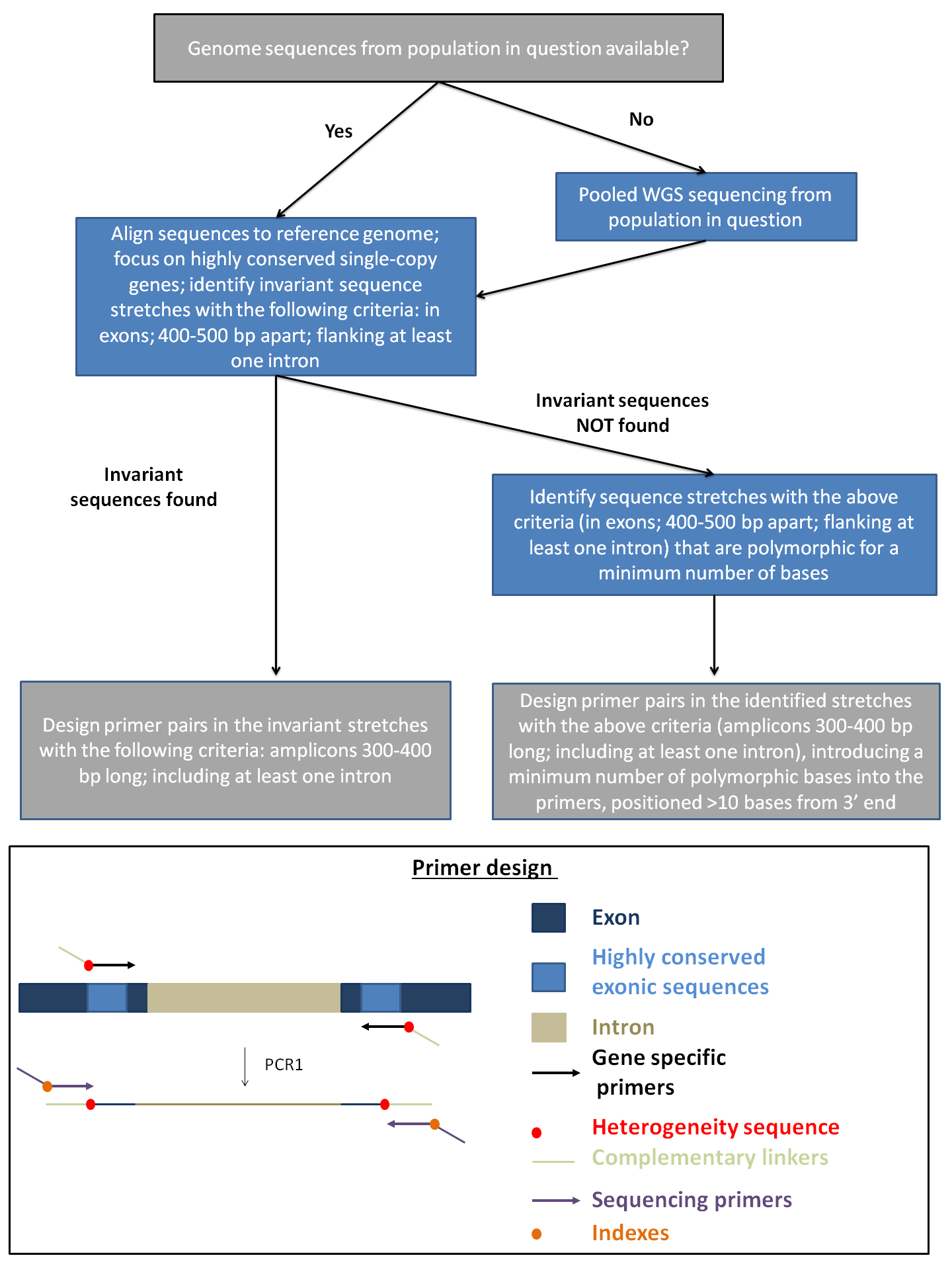

Supplement: Supplementary file 4 — Additional file 4: Figure S1. Strategy for primer design. An outline for choosing suitable primer binding sites and designing primers is shown for different scenarios, along with suggested parameter values for the primers and amplicons. [file 13007_2019_433_MOESM4_ESM.png]
